# Outcome of children with oligoarticular juvenile idiopathic arthritis compared to polyarthritis on methotrexate- data of the German BIKER registry

**DOI:** 10.1186/s12969-021-00522-4

**Published:** 2021-03-22

**Authors:** A. Raab, T. Kallinich, D. Huscher, I. Foeldvari, F. Weller-Heinemann, F. Dressler, J. B. Kuemmerle-Deschner, A. Klein, G. Horneff

**Affiliations:** 1 Department of Pediatric Respiratory Medicine, Immunology and Critical Care Medicine, Children’s university hospital Charité, Augustenburger Platz 1, 13353 Berlin, Germany; 2 Institute of Biometry and Clinical Epidemiology and Berlin Institute of Health, Charité – Universitätsmedizin, Berlin, Germany; 3 Hamburg Centre for Pediatric and Adolescence Rheumatology Centre for Treatment of Scleroderma and Uveitis in Childhood and Adolescence, Hamburg, Germany; 4 Division of Pediatric Rheumatology, Prof. Hess Children’s Hospital, Bremen, Germany; 5 Department of Paediatric and Adolescents medicine, Medical Faculty, University Hospital of Cologne, Cologne, Germany; 6 Division of Pediatric Pneumology, Hannover Medical School, Hannover, Germany; 7 Division of Rheumatology, Department of Pediatrics and autoinflammation reference center Tuebingen, University Hospital Tuebingen, Tuebingen, Germany; 8 Department of Paediatrics, Centre for Paediatric Rheumatology, Asklepios Clinic Sankt Augustin, Sankt Augustin, Germany

**Keywords:** Oligoarticular juvenile idiopathic arthritis, Methotrexate, Outcome, Comparison, Polyarthritis

## Abstract

**Background:**

Oligoarticular juvenile idiopathic arthritis (oligoJIA) is the most commonly diagnosed category of chronic arthritis in children. Nevertheless, there are no evidence- based guidelines for its treatment, in particular for the use of methotrexate (MTX). The primary objective of this analysis is to evaluate the outcomes in patients with persistent oligoJIA compared to those with extended oligoJIA and rheumatoid factor (RF) negative polyarthritis treated with methotrexate.

**Methods:**

Patients with persistent or extended oligoJIA or RF negative PA recorded in the Biologics in Pediatric Rheumatology Registry (BiKeR), receiving methotrexate for the first time were included in the analyses. Efficacy was determined using the Juvenile Arthritis Disease Activity Score 10 (JADAS 10). Safety assessment included the documentation of adverse and serious adverse events.

**Results:**

From 2005 through 2011, 1056 patients were included: 370 patients with persistent oligoJIA, 221 patients with extended oligoJIA and 467 patients with RF negative PA. Therapeutic efficacy was observed following the start of methotrexate.

Over a period of 24 months JADAS-minimal disease activity (JADAS ≤2) was reached in 44% of patients with persistent oligoJIA, 38% with extended oligoJIA, 46% with RF negative PA, JADAS-remission defined as JADAS ≤1 was reached in 33% of patients with persistent oligoJIA, 29% with extended oligoJIA and 35% (RF negative PA). Patients with extended oligoJIA achieved JADAS remission significantly later and received additional biologic disease-modifying drugs significantly more often than patients with persistent oligoJIA or RF negative PA (*p* < 0.001)**.** Tolerability was comparable. New onset uveitis occurred in 0.3 to 2.2 per 100 patient years.

**Conclusions:**

Patients with persistent oligoJIA taking methotrexate are at least as likely to enter remission as patients with extended oligo JIA or polyarticular JIA. Patients with extended oligoJIA achieved JADAS remission significantly later. Within 2 years, almost half of the patients with persistent oligoJIA achieved JADAS-minimal disease activity.

## Background

Oligoarticular juvenile idiopathic arthritis (oligoJIA) is the most commonly diagnosed category of chronic arthritis among children in Europe and North America. It accounts for 50-80% of all children with chronic arthritis [1, 2]. OligoJIA is defined as a chronic inflammatory arthritis of unknown origin that begins before the age of 16 years and persists for longer than 6 weeks [3]. Most of the patients with oligoJIA suffer from the clearly defined typical pediatric form characterized by common features: asymmetric arthritis, early onset (younger than 6 years), female gender, positive antinuclear antibodies (ANA) and high risk for developing uveitis [4]. The International League of Associations for Rheumatology (ILAR) classifications distinguishes between two categories of oligoJIA: persistent, if no more than four joints are affected during the disease course and, or extended, if after the initial 6-months of disease, the total number of affected joints exceeds four [3].

Rheumatoid factor (RF) negative polyarthritis comprises of about 20% of the JIA patients and is also a heterogeneous category with a phenotype very similar to the ANA positive early-onset oligoJIA and another phenotype characterized by symmetric arthritis of large and small joints, onset at school age and negative ANA. The similarity to the first subtype to oligoJIA suggests that it may be the same type of disease except for the number of affected joints [5]. New molecular genetic evidence suggests that the ILAR- classification should be redefined [6]. These latest genetic findings challenge the division between pediatric and adult arthritis [7]. Recently there were efforts to provide a new evidence-based classification instead of the ILAR-classification [4].

In general, patients with oligoJIA at presentation have the best outcome [8, 9]. Nevertheless, they can also bear a great burden of disease. Albers and colleagues found a similar median physician’s global assessment in oligoJIA, extended JIA and RF negative polyarthritis even the cumulative time of active disease was different [9]. Functional disability in oligoJIA is much rarer than in the other subtypes [10], but children with persistent oligoJIA can also develop localized growth abnormalities. Commonly affected is the knee, with the leg on the affected side becoming longer. They also can have musculoskeletal abnormalities, like flexion contractures or atrophic muscles around the inflamed joint [11, 12]. Although oligoJIA is the most common subtype of JIA, and MTX the most frequently used conventional disease-modifying drug (cDMARD) in pediatric rheumatology, there are only few evidence-based treatment guidelines [12]. The main objective of this analysis was to evaluate the outcome of patients with persistent oligoJIA treated with MTX, using data from the German BiKeR registry. With regard to the upcoming new classification criteria for JIA, we compared the data to patients with extended oligoJIA and RF negative polyarthritis.

## Methods

Patients: Data of children and adolescents enrolled in the German BiKeR Registry between 2005 and 2011 were analyzed, the last follow-up was in September 2018. This registry has been described in detail in previous reports [13, 14].

Inclusion criteria were diagnosis of persistent or extended oligoJIA and RF negative polyarthritis according to the ILAR criteria [3]. Initiation of therapy with methotrexate was defined as baseline. Patients already treated with a biologic agent were excluded. Patients on stable treatment with further conventional DAMRDs such as hydroxychloroquine or sulfasalazine were included. Follow-up assessments were after three months, six months, 12 months, 18 months and 24 months. Only patients with at least one follow-up visit were included. Due to the character of the registry, the number of patients observed decreased during the course of the study.

Outcome variables comprised of clinical and laboratory parameters: global assessment of disease activity on a visual analogue scale (VAS) from 0 to 10 (10 highly active), number of tender, swollen, active joints, number of joints with limitation of motion, duration of morning stiffness (minutes), erythrocyte sedimentation rate (ESR) and C-reactive protein (CRP).

Patient-reported outcome measures included overall well-being, rated by parents or patients older than 12 years on a visual analogue scale (VAS, score from 0 (best) to 10 (worst)). The patient’s functional capacity in daily living activities was assessed using the German version of the Childhood Health Assessment Questionnaire (CHAQ, score from 0 (best) to 3 (worst)) [15, 16].

### Efficacy assessment

Treatment efficacy was measured by the Juvenile Arthritis Disease Activity Score (JADAS 10) based on physician’s global assessment of disease activity (VAS), the number of joints with active arthritis, and the patient’s or parent’s global assessment of overall well-being (VAS), and CRP [17]. If the ESR is missed, CRP was used to calculate the JADAS.According to the criteria of Consolaro et al. the cutoff for minimal disease activity was JADAS-10 ≤ 3.8 for polyarthritis and extended oligoJIA and JADAS- 10 ≤ 2 for persistent oligoJIA. For better comparability, we decided to use the same cutoff ≤2 for polyarthritis and oligoJIA. Cut off for remission was JADAS-10 ≤ 1 [17, 18].

Efficacy was analyzed after an observation period of 24 months. Data was captured if methotrexate was discontinued or if a biologic disease-modifying drug (bDMARD) was started.

Effectiveness of the treatment with MTX was defined according the intention-to-treat principle: Patients who discontinued due to inefficacy or intolerance were classified as non- responder.

### Safety assessment

Safety was analyzed based on adverse event (AE) reporting. An AE was defined as any untoward medical occurrence in a subject administered a pharmaceutical product, even without a causal relationship to the treatment. The Medical Dictionary MedDRA V.18.1 was used to code all events. Serious adverse events (SAE) included death, a life-threatening event, an event leading to or prolonging hospitalization, persistent or significant disability/incapacity or an important medical event requiring medical or surgical intervention to prevent a serious outcome or congenital anomaly or birth defect. Rates of AE were calculated per 100 patient years with 95% intervals.

### Statistical analysis

For continuous variables, means and standard deviations (SD) or median and interquartile range (IQR), and for categorical variables counts and percentages are shown. Group comparisons for continuous variables were conducted with the t-test or Kruskal-Wallis-test with post-hoc adjusted group-wise comparisons. Time to MTX discontinuation was evaluated by Kaplan-Meier analysis. Differences in JADAS development were analyzed with linear mixed models. Since adverse events (AE) and methotrexate discontinuations did occur multiple times for certain patients, calculation of AE rates were calculated based on the patient years under observation, and reasons for MTX discontinuation were referred to the total number of MTX discontinuations. The Wald test was used for comparison.

## Results

### Patient characteristics

From 2005 through 2011 1058 patients with persistent or extended oligoJIA and RF negative polyarthritis from 28 centers were enrolled in the German BIKER registry. The characteristics of patient groups at baseline are summarized in Table 1. At baseline, gender, age at onset, age when starting MTX significantly differed between the three subtypes, mostly triggered by differences of the polyarthritis cohort compared to the two oligoJIA groups, while age at onset differed between all three groups. ANA was present significantly more frequent in patients with extended oligoJIA, while HLA-B27 was similarly present in all three groups (8.9-12.8%).

**Table 1:** Patient characteristics at baseline (start of methotrexate)

	persistent oligoJIA	extended oligoJIA	RF negative polyarthritis
patients [n]	370	221	467
Female gender [n, %]	244 (66)	161 (73)	370 (79)
age at onset [years]			
mean, +/− SD	6.4 +/− 4.3	5.2 +/− 3.7	8.4 +/− 4.7
median, (IQR)	5.0 (2.6; 9.75)	4.2 (2.2;7.4)	8.9 (4.03;12.4)
age at MTX start [years]			
mean, +/− SD	8.7 +/− 4.7	8.5 +/− 4.3	9.7 +/− 4.9
median, (IQR)	8.1 (4.5;12.7)	8.6 (0.6;4.4)	10.3 (5.7;14)
disease duration [years]			
mean, +/− SD	2.4 +/− 2.9	3.2 +/− 3.7	1.3 +/− 2
median, (IQR)	1.1 (0.5;3.1)	1.6 (0.64;4.41)	0.5 (0.26;1.44)
MTX starting dose [mg/m2]			
median, (IQR)	12.6 (11;14.1)	12.1 (10.5;13.8)	12.5 (10.9;14)
Pretreatment			
NSAR [n, %]	315 (85.1)	195 (88.2)	394 (84.4)
i.a. Steroids [n, %]	186 (50.3)	85 (38.46)	100 (4.28)
concomitant treatment at enrolment, [n, %]			
oral steroids [n, %]	48 (13)	33 (15)	147 (32)
steroids pulse therapy last 3 month [n, %]	6 (1.6)	3 (1.4)	20 (4.3)
conventional DMARDs, [n, %]	9 (2.4)	9 (4.1)	10 (2.1)
ANA- positive [n, %]	216 (58.49)	146 (66.1)	240 (51.4)
Uveitis at enrolment [n, %]	49 (13.2)	27 (12.2)	9 (1.9)
Active arthritis at enrolement [n, %]	329 (88.9)	209 (94.6)	443 (94.9)
number of active joints			
mean +/−SD	2.1 +/− 1.8	4.1 +/− 4.1	11.1 +/− 10.3
median (IQR)	3 (2; 20)	5 (3; 34)	15 (8; 55)
ESR > 20 mm/1 h [n, %]	128 (37.2)	83 (41.9)	197 (45.4)
CRP > 5 mg/l [n, %]	117 (33.7)	91 (45.0)	217 (48.5)
JADAS 10 mean, +/−SD [0–14] median (IQR)	9.9 +/− 4.8 9.9 (6.3; 13.2)	11.8 +/− 4.9 11.8 (8.5; 15.2)	16.7 +/− 6.3 21.2 (17.0; 21.2)

*DMARD* biologic disease-modifying antirheumatic drug, *MTX* methotrexate, *ESR* erythrocyte sedimentation rate, *CRP* C-reactive protein, *JADAS* juvenile Disease activity Score (JADAS 10, mean SD, median IQR), LOM limitation of motion, number of active joints (defined by the presence of swelling or, when absent, the limited range of motion accompanied by either pain or tenderness on motion)

Compared to RF negative polyarthritis, uveitis occurred significantly more frequent in patients with persistent and extended oligoJIA (*p* < 0.001).

The number of swollen and active joints differed significantly across all three groups, JADAS 10 as well as the JADAS versions including ESR or CRP also differed significantly across all three subtypes; ESR and CRP were significantly higher in patients with polyarthritis, ESR also only when compared to persistent oligoJIA.

### Treatment

MTX starting dosage ranged from 12.1 to 12.6 mg/m^2^ weekly and did not differ between the cohorts. 57% / 53% / 51% of patients initially received oral MTX in persistent /extend OJIA and polyarthritis cohort.

### Concomitant treatment

At baseline none of the patients received a biological disease-modifying antirheumatic drug (bDMARD). Patients with RF negative polyarthritis were initially significantly more frequently treated with oral glucocorticosteroids (*p* < 0.001) and steroid pulse therapy (p=0.024). Patient with extended oligoJIA received additional DMARDs at baseline more frequently (4% vs. 2%), while at 24 month 6-7% received additional DMARDs in all three diagnosis groups. A bDMARD was started in 49 (13%) of patients with persistent oligoJIA (Etanercept in 30 (8.1%), Adalimumab in 18 (4.9%) and Infliximab in 1 (0.3%)), in 102 patients (46.2%) in the extended oligoJIA (Etanercept in 80 (26.2%), Adalimumab in 18 (8.1%), Abatacept in 2 (0.9%), Tocilizumab in 2 (0.9%)), and in 172 (36.8%) in the RF negative PA cohort (Etanercept in 133 (28.5%), Adalimumab in 35 (7.5%), Abatacept in 1 (0.2%), Tocilizumab in 2 (0.4%) and Infliximab in 1 (0.2%)). Significantly less patients in the persistent oligoJIA started a biologic agent (RR 0.29 (0.21-0.38); *p* < 0.0001 compared to extended oligoJIA; RR 0.36 (0.27-0.48); *p* < 0.0001 compared to RF negative PA).

### Efficacy

Regardless of the JIA category, patients showed significant improvement in the number of active joints, duration of morning stiffness, physician‘s and parent‘s global assessment at the follow-up visits after 3, 6, 12, 18 and 24 months (*p* < 0.001). Apart from parent’s global assessment, significantly different developments over time were observed for the three diagnosis groups (*p* < 0.001). Physician’s global assessment (median IQR), active joints (median IQR) and CRP > 5 (n, %) are shown in Table 2 for all three groups over two years.

**Table 2:** Selected effectiveness parameters after three months, six months, 12 months, 18 months and 24 months

	Month 0	Month 3	Month 6	Month 12	Month 18	Month 24
Physician global assessment; median (IQR)						
persistent oligoJIA	40 (20; 63)	7 (2; 17)	3 (1; 13)	3(1;13.5)	3 (0; 13)	2 (0; 8)
extended oligoJIA	45 (25; 65)	12 (4; 23)	8.5(2,5; 20,5)	3(1; 13.5)	3 (0; 13)	3 (0; 11)
RF negative PA	54.5 (34; 79)	15 (5; 29)	9 (3; 20)	5 (1; 16)	3 (0; 10)	3 (0; 7)
parents/patient global assessment; median (IQR)						
persistent oligoJIA	35 (11; 55)	6 (2; 19)	4 (1; 13)	3 (0, 11)	2 (0, 9)	2 (0; 10)
extended oligoJIA	36 (15; 55)	12(2; 29)	7 (2; 18)	5 (1,18)	3 (0; 17)	2 (0, 16)
RF negative PA	44 (22; 63)	14 (4; 31)	8 (2; 22)	6 (1; 19)	3 (0; 19)	3 (0; 11)
CRP > 5; n (%)						
persistent oligoJIA	117 (33.7)	27 (13.6)	20 (11.1)	9 (5.4)	10 (6.6)	10 (8.1)
extended oligoJIA	91 (45)	26 (20.6)	21 ((16.7)	17 (14.5)	12 (12.1)	11 (11.6)
RF negative PA	217 (48.5)	53 (18.3)	53 (18.7)	44 (17.5)	22 (10.5)	18 (10.2)

Data as observed. #Data are given as median and interquartile range (IQR) of patients on treatment as observed

100 mm scale was used to assess the patient’s and physician’s global assessment of disease activity (0 = best, 100 = worst) number of active joints (defined by the presence of swelling or, when absent, the limited range of motion accompanied by either pain or tenderness on motion)

**Fig. 1:**
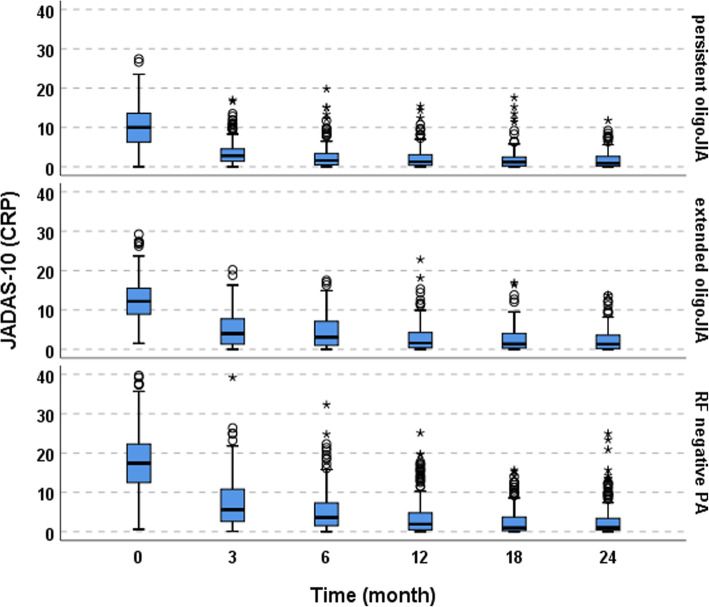
JADAS- 10 and active joints over time^a^. JADAS- 10 Juvenile Disease Activity Score, CRP C-reactive protein, oligoJIA oligoarticular juvenile idiopathic arthritis, RF negative PA rheumatoid factor negative polyarthritis. ^a^For better visibility, the y-axis of active joints was cut off at 35; by that for RF negative PA, 18 cases with 36–55 active joints at month 0 and 1 case with 44 active joints at month 18 were omitted. ° outliers. *extreme values

**Fig. 2:**
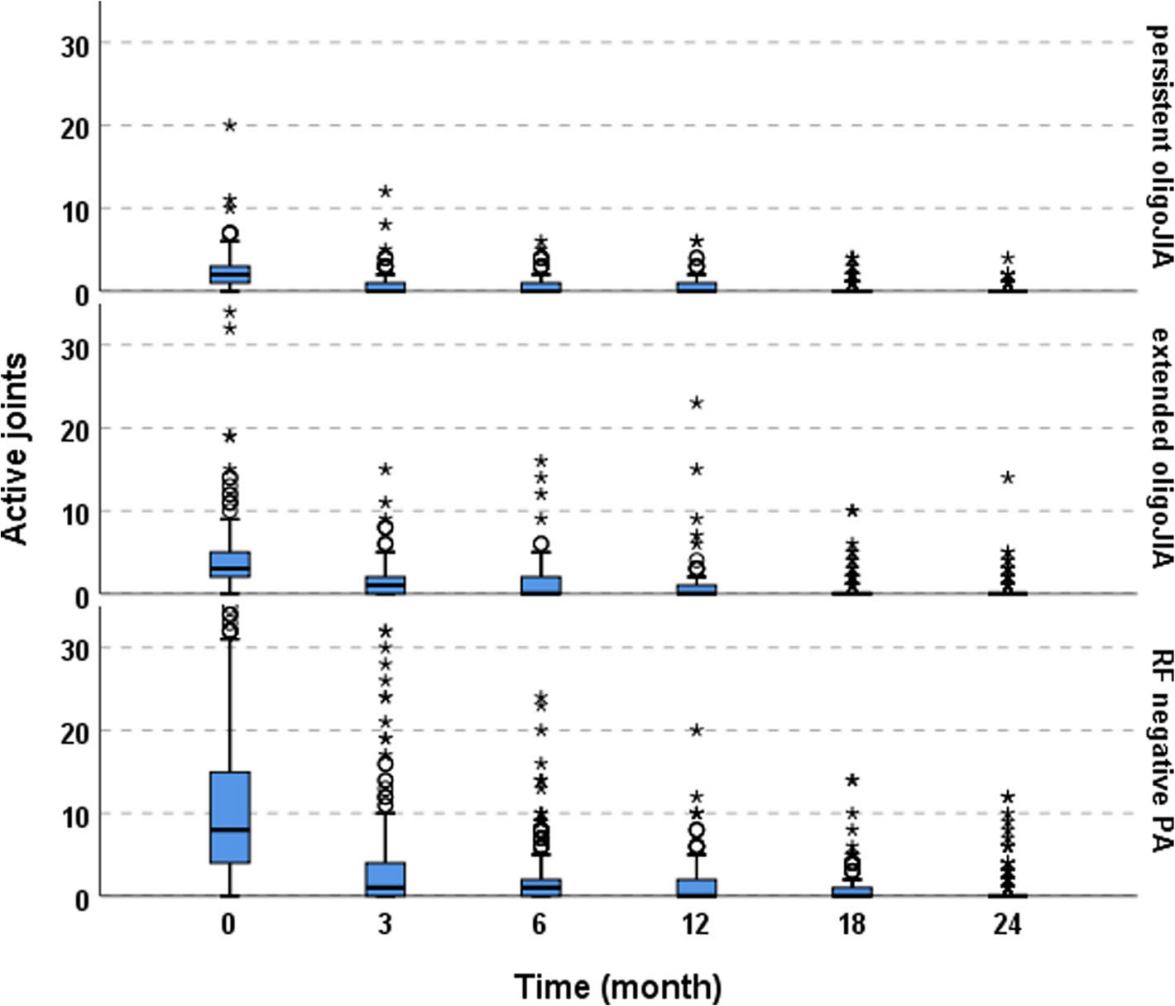
JADAS- 10 and active joints over time^a^. JADAS- 10 Juvenile Disease Activity Score, CRP C-reactive protein, oligoJIA oligoarticular juvenile idiopathic arthritis, RF negative PA rheumatoid factor negative polyarthritis. ^a^For better visibility, the y-axis of active joints was cut off at 35; by that for RF negative PA, 18 cases with 36–55 active joints at month 0 and 1 case with 44 active joints at month 18 were omitted. ° outliers. *extreme values

**Fig. 3:**
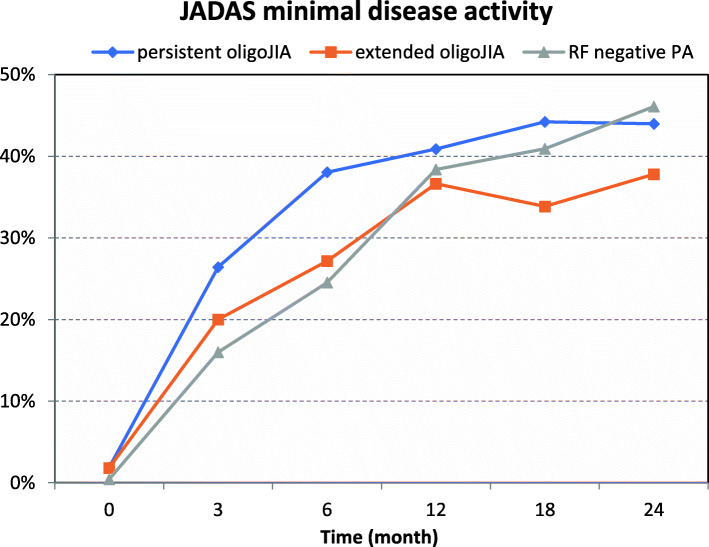
JADAS minimal disease activity and JADAS remission. JADAS minimal disease activity (defined as JADAS ≤2) and JADAS-remission (defined as JADAS ≤1) in patients with persistent oligoJIA, extended oligoJIA and RF negative polyarthritis (PA) at baseline and follow up assessment after three months, six months, 12 months, 18 months and 24 months

In all three JIA categories, JADAS 10 decreased over timewhile patients with RF negative polyarthritis had higher JADAS values at MTX initiation (*p* < 0.001), they showed a significantly higher decline of JADAS in the first months of therapy (*p* < 0.001) (Fig. 1). Figure 2 demonstrates the decrease in the number of active joints during the first two years. After two years almost half of the patients with persistent oligoJIA had reached JADAS minimal disease activity (Fig. 3).

The results of the efficacy analysis showed no difference between ANA positive and ANA negative patients (data not shown). Thus according to our analyses we could not see that ANA positivity has an influence on the MTX response.

**Fig. 4:**
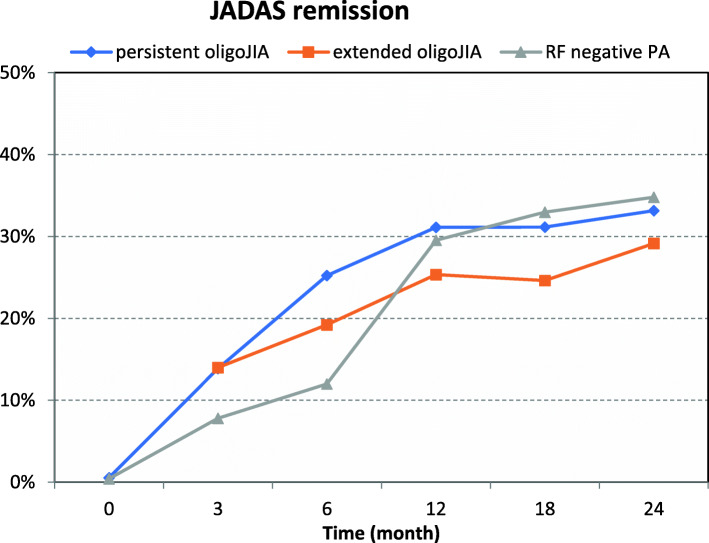
JADAS minimal disease activity and JADAS remission. JADAS minimal disease activity (defined as JADAS ≤2) and JADAS-remission (defined as JADAS ≤1) in patients with persistent oligoJIA, extended oligoJIA and RF negative polyarthritis (PA) at baseline and follow up assessment after three months, six months, 12 months, 18 months and 24 months

After one year, about 30% of the patients with persistent oligoJIA and RF negative polyarthritis were in remission (Fig. 4). Patients with extended oligoJIA had lower remission rates at 3 and 6 months.

### Safety

#### Adverse effects and discontinuation

During about 2621 patient years of treatment, there were 982 reports of adverse events and 29 of serious adverse events. Most adverse events (AE) were documented in the 467 patients with polyarticular JIA. The lowest rate of AE has been reported in patients with oligoJIA. The rate for adverse events was significantly lower in persistent oligoJIA patients than in both patient cohorts. The most commonly reported adverse events were infections, followed by elevated transaminases and JIA- reactivation (Table 3).

**Table 3:** Safety

	persistent oligoJIA		extended oligoJIA		RF negative PA	
	*n* = 370 738.01 PY	Rate per 100 patient years (95%-CI)	*n* = 221 753.54 PY	Rate per 100 patient years (95%-CI)	*n* = 467 1129.63 PY	Rate per 100 patient years (95%-CI)
AE, total number	194	26.2(22.8–30.2)	247	32.7(28.9–37.1)	541	47.8(44.0–52.1)
infections, total number	26	3.5(2.4–5.2)	60	7.9(6.1–10.2)	143	12.6(10.7–14)
SAE infections	2	0.3(0.07–1.9)	1	0.1(0.02–0.9)	3	0.3(0.09–0.8)
JIA-Reactivation	3	0.4(0.1–1.2)	6	0.7(0.3–1.7)	8	0.7(0.3–1.4)
neutropenia	3	0.4(0.1–1.2)	0		3	0.2(0.08–0.8)
thrombocytopenia	0		0		0	
elevated transaminases	37	5.0(3.6–6.9)	38	5.0 (3.6–6.9)	68	6.0(4.7–7.6)
hair loss	1	0.1(0.01–0.96)	1	0.1(0.01–0.9)	6	0.5(0.2–1.1)
uveitis	16	2.2(1.2–3.5)	15	2.0(1.1–3.3)	4	0.3(0.1–0.9)

*AE* adverse event, *SAE* serious AE, *MAS* macrophage activation syndrome, *PY* patient years

The Wald test was used for comparison

There were no reports on macrophage activation syndrome, hypersensitivity or thrombocytopenia.

Rates and reasons for MTX discontinuation are demonstrated in Table 4. Remission was the most common reason for drug discontinuation in all JIA categories, followed by discontinuation on patients’ demand and drug intolerance.

**Table 4:** Rates and reasons for methotrexate discontinuation (several reasons could be given in parallel)

	persistent oligoJIA	extended oligoJIA	RF negative PA
Discontinuations total [n, %]	198(100)	317 (100)	564 (100)
Inefficacy [n, %]	13 (6.8)	19 (6.2)	27 (5.0)
Intolerance [n, %]	45 (23.2)	97 (31.6)	177 (32.8)
Remission [n, %]	118 (61.5)	162 (52.8)	289 (53.1)
Others [n, %]	17 (8.9)	29 (9.7)	62 (11.6)
Patients demand [n, %]	79 (41.1)	134 (43.4)	231 (42.9)

## Discussion

Although oligoJIA is the most common JIA category and MTX is the standard treatment drug in JIA, there are scarce data on the use of MTX in patients with oligoJIA. The German BiKeR registry is one of the largest national registries on the use of biologics. It includes a control group of patients who at baseline had not been treated with a biologic but started treatment with MTX and were prospectively followed. Due to the design of our analysis, all patients were treated with MTX so there is no control group. Nevertheless, in a large number of patients it could be demonstrated that patients with oligoJIA treated MTX are at least as likely to enter remission as patients with extended oligoJIA or RF negative polyarthritis with regard to target such as JADAS minimal disease activity or JADAS remission. Within two years almost half of the patients reached JADAS minimal disease activity (Fig. 3). Significantly less patients were treated with bDMARDs during the later course of the disease which can in part be explained by the lack of approval of biologics for persistent oligoJIA.

Moreover, tolerability was superior with regard to the total number of adverse events reported to the registry (Table 3) although dosing was comparable to the patients with extended JIA and RF negative Polyarthritis (Table 1). MTX was overall well- tolerated and the vast majority of patients in the study did well. While there was no difference in the occurrence of adverse events of special interest, the number of uveitis flares was significantly higher in the persistent and oligoJIA cohort than in the RF negative polyarthritis.

In a systemic literature review ANA positivity was found to be a predictor of better response three other studies [19]. In our analyses we could not see that ANA positivity had an influence on the MTX response.

Our figures are also largely comparable to older outcome cohorts such as the Research on Arthritis in Canadian Children emphasizing Outcome study (ReACCH- Out) and the Inception Cohort of Newly diagnosed patients with juvenile idiopathic arthritis (ICON) [20, 21]. One recently published first treat-to-target study in patients with JIA also showed similar results: After 24 months inactive disease was achieved by > 70% of patients. Due to the small number of cases with oligoJIA [11], the interpretation of these results is limited [22].

In our analyses patients with RF-negative polyarthritis started with higher JADAS scores at MTX initiation, but showed a significantly higher decline of the JADAS in the first months of therapy, reaching similar scores after 18 months as persistent and extended oligoJIA. Ravelli and colleagues described the extended oligoJIA subtype as the best predictor of MTX efficacy [23]. In our data patients with extended oligoJIA achieved remission significantly later, even all of them were treated with MTX. Also, in the ICON cohort the longest time from diagnosis to inactive disease was observed in extended oligoJIA [21]. A long-term Nordic cohort study observed a similar remission outcome or even worse than those of patients with RF-negative PA [9, 24]. As far as we know, it is not clear yet, why patients with extended oligoJIA achieve remission later, but one possible reason for that could be the lack of treat to target strategies for these patients and due to that a later initiation of early aggressive therapy. As described above in our cohort patients with RF negative polyarthritis were initially significantly more frequently treated with glucocorticosteroids. New molecular genetic insights suggests that oligoJIA and RF negative polyarthritis may be the same disease. Also previous investigations have shown that ANA positive patients with persistent or extended oligoJIA seem to have the same disease characteristics and only differ in the number of affected joints [25, 26]. There are first efforts to create new evidence- based classification criteria of JIA [4]. These new findings and new classification may improve therapy and outcome also of the extended oligoJIA.

Although MTX is the most widely used cDMARD in pediatric Rheumatology, there are almost no consensus-based recommendations for the use of MTX in oligoJIA [27, 28]*.* In a multicenter, prospective, randomized open-label trial Ravelli et al. investigated the effectiveness of methotrexate plus intra-articular corticosteroids in oligoarticular JIA. The study suggests that additional therapy with MTX does not improve the efficacy of intra-articular corticosteroid therapy [12]. In a small randomized, placebo- controlled, crossover trial of methotrexate in patients with extended oligoarthritis (43) MTX was found to be an effective treatment [29]. Birk et al. studied prospectively 19 children with oligoarthritis and also concluded, that MTX seems to be very effective treatment for children with oligoarticular JIA [30]. Further trials are needed.

Lately there are approaches to create recommendations like the PICO (population, intervention, comparator, outcome) research questions and recommendations from the MARAJIA (Methotrexate Advice and RecommendAtions on Juvenile Idiopathic Arthritis) Expert Meeting in Italy. MTX is recommended as the first-line treatment in oligoJIA that persists despite Nonsteroidal anti-inflammatory drugs (NSAIDs) and intraarticular steroids therapy (Grade of evidence 1A) [31].

## Conclusions

Patients with persistent oligoJIA taking methotrexate are at least as likely to enter remission as patients with extended oligo JIA or polyarticular JIA. Further evaluation of a large patient cohort with JIA on methotrexate is important to develop treat-to-target strategies for patients with oligoJIA as they already exist for patients with polyarthritis.

## Data Availability

Not applicable.

## Abbreviations

AE: Adverse Event
ANA: Antinuclear antibodies
bDMARD: Biological disease- modifying drug
BIKER: Biologics in Pediatric Rheumatology Registry
CHAQ: Childhood Health Assessment Questionnaire
RP: C- reactive protein
csDMARD: Conventional disease-modifying drug
DMARD: Disease-modifying antirheumatic drug
ESR: Erythrocyte sedimentation rate
ILAR: International League of Association for Rheumatology
IQR: Interquartile range
JADAS: Juvenile Disease Activity Score
JIA: Juvenile idiopathic arthritis
MARAJIA: Methotrexate Advice and RecommendAtions on Juvenile Idiopathic Arthritis
MDA: Minimal disease activity
MTX: Methotrexate
NSAIDs: Nonsteroidal anti-inflammatory drugs
oligoJIA: Oligoarticular juvenile idiopathic arthritis
PICO: Population, intervention, comparator, outcome
SAE: Serious adverse event
SD: Standard deviation
VAS: Visual analogue scale
